# 

**Published:** 1891-12

**Authors:** 


The eighty-sixth annual meeting of the Medical Society of the State of New York will be held at Albany, Tuesday, Wednesday, and Thursday, February 2, 3, and 4, 1892. Dr. Seneca D. Powell, No. 12 West 40th street, New York, Dr. James D. Spencer, of Watertown, and Dr. Franklin Townsend, No. 2 Park place, Albany, have been appointed the Business Committee. Any communications regarding papers, or any matter pertaining to the business of the Society, which may properly come before the Business Committee, should be addressed to Dr. Seneca D. Powell, Chairman, 12 West 40th street, New York City.

A. WALTER SUITER,

*President.*

F. C. Curtis,

*Secretary.*

### BOOKS AND PAMPHLETS RECEIVED.

A Text-book of Physiology. By M. Foster, M. D., LL. D., F. R. S., Professor of Physiology in the University of Cambridge, England. Fourth American, from the fifth English edition, thoroughly revised. Octavo, 1072 pages, 282 engravings. Cloth, $4.50; leather, $5.50. Philadelphia: Lea Brothers & Co. 1891.

A Practical Treatise on the Diseases of Women. By T. Gaillard Thomas, M. D., LL. D., Emeritus Professor of Diseases of Women in the College of Physicians and Surgeons, New York, and Paul F. Mundé, M. D., Professor of Gynecology in the New York Polyclinic. New (sixth) edition, thoroughly revised and rewritten by Dr. Mundé. In one large and handsome octavo volume of 824 pages, with 347 illustrations. Cloth, $5.00; leather, $6.00. Philadelphia: Lea Brothers & Co. 1891.

Ptomaines and Leucomaines and Bacterial Proteids; or, The Chemical Factors in the Causation of Disease. By Victor C. Vaughan, Ph. D., M. D., Professor of Physiological and Pathological Chemistry, and Associate Professor of Therapeutics and Materia Medica, in the University of Michigan, and Frederick G. Novy, M. D., Instructor in Hygiene and Physiological Chemistry in the University of Michigan. New edition. In one handsome 12mo volume of 389 pages. Cloth, $2.25. Philadelphia: Lea Brothers & Co. 1891.

Manual of Chemistry. A Guide to Lectures and Laboratory Work for Beginners. A Text-book especially adapted for Students of Pharmacy and Medicine. By William Simon, Ph. D., M. D., Professor of Chemistry and Toxicology in the College of Physicians and Surgeons, Baltimore, and Professor of Chemistry in the Maryland College of Pharmacy. New (third) edition. In one octavo volume of 477 pages, with forty-four wood-cuts and seven colored plates, illustrating fifty-six of the most important chemical tests. Cloth, $3.25. Philadelphia: Lea Brothers & Co. 1891.

The Comparative Anatomy of the Domesticated Animals. By A. Chauveau, M. D., LL. D., Member of the Institute (Academy of Sciences); Inspector-General of Veterinary Schools in France; Professor of the Museum of Natural History, Paris. Revised and enlarged, with the coöperation of S. Arloing, Director of the Lyons Veterinary School, Professor of Experimental and Comparative Medicine at the Lyons Faculty of Medicine. Second English edition, translated and edited by George Fleming, C. B., LL. D., F. R. C. V. S., Late Principal Veterinary Surgeon of the British Army; Foreign Corresponding Member of the Société Royal de Medicine, and the Société Royal de Medicine Publique, of Belgium; Foreign Associate of the Société Centrale de Medicine Veterinaire of France; Honorary Life Member of the Royal Agricultural Society of England; Foreign Member of the Société Nationale d'Agriculture of France, etc.; Examiner in Anatomy for the Royal College of Veterinary Surgeons. With 585 illustrations. New York: D. Appleton & Co. 1891.

Essentials of Nervous Diseases and Insanity: Their Symptoms and Treatment. A Manual for Students and Practitioners. By John C. Shaw, M. D., Clinical Professor of Diseases of the Mind and Nervous System, Long Island College Hospital Medical School; Consulting Neurologist to St. Catherine's Hospital, and Long Island College Hospital; formerly Medical Superintendent King's County Insane Asylum. Forty-eight original illustrations, mostly selected from the author's private practice. Philadelphia: W. B. Saunders, 913 Walnut street. 1891.

. Annual Report of the State Board of Charities for the Year 1890. Transmitted to the Legislature, February 5, 1891. Albany: James B. Lyon, State Printer. 1891.

Bureau of Education, Circular of Information No. 1, 1891. Contribution to American Educational History. Edited by Herbert B. Adams. No. 10. Higher Education in Indiana. By James Albert Woodburn, Ph. D., Professor of American History in the Indiana University; Sometime Fellow in History, John Hopkins' University. Washington: Government Printing Office. 1891.

Addresses, Papers and Discussions, in the Section of State Medicine, at the Forty-second Annual Meeting of the American Medical Association, at Washington, D. C., May 5 to 8, 1891. Chicago: Printed at the office of the Association. 1891.

Addresses, Papers and Discussions, in the Section of Otology and Laryngology, at the Forty-second Annual Meeting of the American Medical Association, at Washington, D. C., May 5 to 8, 1891. Chicago: Printed at the office of the Association. 1891.

Post-Partum Douche (showing new instrument). By Edwin Pynchon, M. D. Read before the Chicago Medical Society, September 7, 1891.

An Abstract of the Symptoms, with the Latest Dietetic and Medicinal Treatment, of Various Diseased Conditions. The Food Products. Digestion and Assimilation. The New and Valuable Preparations manufactured by Reed & Carnrick, 447 and 449 Greenwich street, New York.

A Clinical Report of Operative Surgery in the Service of Dr. William T. Bull. At the New York Hospital during October and November, 1889, and from February to June, 1890. By Wm. B. Coley, M. D., Late House Surgeon. Reprint: *New York Medical Journal* for April 18 and 25, May 2 and 30, June 20, and August 29, 1891.

Addresses, Papers and Discussions, in the Section of Oral and Dental Surgery, at the Forty-second Annual Meeting of the American Medical Association, at Washington, D. C., May 5 to 8, 1891. Chicago: Printed at the office of the Association. 1891.

Experiments and Researches on Trap Siphonage, Showing the Comparative Merits of the Principal Appliances used for Trap-Seal Protection. By James M. Denton, M. E., Professor of Experimental Mechanics in Stephens' Institute of Technology, Hoboken, N. J. Presented at the Eighteenth Annual Meeting of the American Public Health Association, Charleston, S. C., December 16-19, 1890. Reprint from Vol. XVI. of the Transactions of the American Public Health Association.

Bald Heads. By Albert E. Carrier, M. D., Detroit, Mich. Reprint: Transactions of Michigan State Medical Society. 1891.

Bulletin of the Buffalo Society of Natural Sciences. Buffalo: Reinecke & Zesch, 500 Main street. 1891.

Advantages of Mixed Narcosis in Gynecological Surgery. By J. C. Reeve, M. D., Dayton, Ohio. Reprint: Transactions of the American Gynecological Society, September, 1891.

Cornell University Agricultural Experiment Station. Bulletin 32. Horticultural Division. October, 1891. Tomatoes Published by the University, Ithaca, N. Y. 1891.

A Report of Ten Selected Cases on Laparatomy, with Remarks. By John H. McIntyre, A. M., M. D., of St. Louis, Mo. Reprint: *The Journal of the American Medical Association.*

Case of Gonorrheal Pyosalpinx. Rare Source of Infection (with plate 1). By Z. H. Evans, M. D., Bay City, Mich. Reprint: *Annals of Gynecology and Pediatry,* August, 1891.

The Statistics and Lessons of Fifteen Hundred Cases of Refraction. By George M. Gould, M. D., Ophthalmologist to the Philadelphia Hospital, Philadelphia, Pa. Read in the Section on Ophthalmology, at the Forty-second Annual Meeting of the American Medical Association, held in Washington, D. C., May, 1891. Reprint: *Journal of the American Medical Association,* September, 1891.

Hypnotism versus Morphinism. By William Lee Howard, M. D., Baltimore, Md. Reprint: *Baltimore, Medical and Surgical Record,* June and July, 1891.

The Twenty-second Annual Report of the President of the Inebriates' Home, Fort Hamilton, N. Y., for the year 1889. Brooklyn: 1890.

On the Treatment of Diphtheria in America. By A. Jacobi, New York. Sonderabdruck aus den Verhandlungen des X. Internationalen Medicinischen Congresses. Berlin: Gedruckt bei L. Schumacher.

Extracranial Aneurism. By A. Jacobi, New York. Sonderabdruck aus den Verhandlungen des X. Internationalen Medicinischen Congresses. Berlin: Gedruckt bei L. Schumacher. 1891.

On Dermatol, a Proposed Substitute for Iodoform. Its Use in Surgical Practice. By Charles A. Powers, M. D., Surgeon to the Out-patient Department, New York Hospital; Instructor in Surgery, New York Post-Graduate Medical School and Hospital. Reprint: *The Medical Record,* October 17, 1891.

Some Facts every Practitioner Ought to Know about Squint. By Alfred Rufus Baker, M. D., Cleveland.

### HEALTH BULLETINS.

Michigan State Board of Health. October, 1891.

Meeting of the Michigan State Board of Health. October, 1891.

Progress in Public Health Work in Michigan. November 9, 1891.

Newport, R. I., *Health Bulletin.* Report of Deaths and Contagious Diseases. October, 1891.

New York State Board of Health. September, 1891.

State Board of Health of Tennessee. October, 1891.

Abstract of Sanitary Reports, U. S. Marine Hospital Service. Vol. VI. Nos. 43, 44, 45, 46, and 47.

WANTED—The Berlin Royal Library wishes to complete its file of the Buffalo Medical and Surgical Journal and advertises for Vols. I. to XXVIII. inclusive, of the new series, and the fifteen volumes of the old series. All inquiries on the subject of back numbers and prices should be addressed to the Managing Editor, 284 Franklin street, Buffalo, N. Y.

